# Implementing an intervention to facilitate early detection of deterioration in aged care residents: process evaluation of the EDDIE + trial

**DOI:** 10.1186/s13012-026-01484-5

**Published:** 2026-02-16

**Authors:** Ella L. Bracci, Michelle J. Allen, Hannah E. Carter, Elizabeth Cyarto, Trudy Dwyer, Alison Farrington, Nicholas Graves, Xing J. Lee, Claudia Meyer, Florin Oprescu, Jeffrey Rowland, Carla Shield, Nicole White, Gillian Harvey

**Affiliations:** 1 Caring Futures Institute, College of Nursing and Health Sciences, Flinders University, Adelaide, South Australia Australia; 2 Australian Centre for Health Services Innovation and Centre for Healthcare Transformation, School of Public Health and Social Work, Faculty of Health, Queensland University of Technology, Brisbane, Queensland Australia; 3 School of Public Health and Social Work, Queensland University of Technology, Brisbane, Queensland Australia; 4 Appleton Institute, Central Queensland University, Queensland, Australia; 5 Duke-NUS Postgraduate Medical School, National University of Singapore, Singapore, Singapore; 6 Bolton Clarke Research Institute, Forest Hill, Victoria, Australia; 7 School of Health, University of the Sunshine Coast, Maroochydore, Queensland Australia; 8 Rehabilitation, Ageing and Independent Living (RAIL) Research Centre, Monash University, Frankston, VIC Australia; 9 Faculty of Medicine, University of Queensland, Herston, Queensland Australia; 10 The Prince Charles Hospital, Chermside, Queensland Australia

**Keywords:** Residential aged care, Early detection, Clinical Deterioration, Avoidable Hospitalisations, Process evaluation, i-PARIHS

## Abstract

**Background:**

EDDIE + was a stepped wedge cluster randomised controlled trial with an embedded process evaluation in 11 residential aged care (RAC) homes in Queensland, Australia. The intervention aimed to upskill RAC staff to identify and manage deterioration to reduce unnecessary hospital transfer through education, decision support tools, diagnostic equipment and local facilitation. Main trial results indicated 46% of hospital admissions were due to falls and no significant improvements to outcome measures including hospital bed days were achieved. These findings were examined through a process evaluation.

**Methods:**

A mixed methods approach guided by the i-PARIHS framework was used to assess fidelity, acceptability, mechanisms of impact and feasibility of implementation, including barriers and enablers. Semi-structured interviews, self-efficacy surveys, and project tracking documents were used. Qualitative data were coded to the i-PARIHS framework and quantitative data were analysed using linear mixed modelling.

**Results:**

Fidelity varied considerably due to workforce shortages including vacancy in the local clinical facilitator role, high workload, COVID-19, and other contextual factors. Differences in job and team-related staff self-efficacy before and after the introduction of EDDIE + were not statistically significant. However, inductive thematic analysis of the questionnaires indicated that staff felt their knowledge, skills, confidence and communication had increased.

**Conclusions:**

The process evaluation indicates high acceptability of the EDDIE + intervention. However, fidelity and intended mechanisms of impact were mixed despite substantial pre-planning prior to implementation. For future studies, specific barriers in the RAC setting such as staffing and turnover may be unable to be adequately addressed without systemic change.

**Trial registration:**

The trial is prospectively registered with the Australia New Zealand Clinical Trial Registry (ACTRN12620000507987, registered 23/04/2020).

**Supplementary Information:**

The online version contains supplementary material available at 10.1186/s13012-026-01484-5.

## Key Points

• The EDDIE+ intervention had high acceptability from RAC staff who felt their skills and confidence were increased.

• There were health system level and workforce level barriers such as staff shortages and COVID-19 that led to variation in fidelity and ability to deliver the EDDIE+ intervention across homes.

• Opportunities for RAC staff to upskill and reduce unnecessary hospital transfers through identification and response to deterioration remains a key priority in this setting.

**Supplementary Information:**

The online version contains supplementary material available at 10.1186/s13012-026-01484-5.

## Background

Unplanned hospital transfers and admissions from residential aged care (RAC) homes contribute to a considerable proportion of hospital bed days and healthcare costs [1]. Almost 50,000 cases of non-admitted emergency department presentations from RAC homes occurred during 2020–2021, equating to a cost of AUD$112 million [1]. Research suggests that some hospital transfers from RAC homes are preventable and that they increase the risk of mortality and in-hospital complications [2–4]. Reducing the need for hospital transfer where appropriate is an important objective not only from a health system perspective, but from the perspective of the residents themselves, who may experience considerable stress and prefer to stay within their home setting.

To achieve this objective, staff in RAC homes need the appropriate knowledge, skills and capacity to identify and manage clinical deterioration. Previous research has evaluated interventions for upskilling staff in managing clinical deterioration. For example, the US study, ‘Interventions to Reduce Acute Care Transfers’ (INTERACT) was a cluster randomised controlled trial involving 85 homes that aimed to determine the effect of training and support on hospital admissions and emergency department visits [5]. RAC staff were provided with tools to recognise and evaluate a change in a resident’s condition and information on alternative care pathways and advance care planning [5]. The intervention did not have a statistically significant effect on all-cause hospitalisations, considered avoidable, 30-day hospital readmissions and ED visits [5]. The Better Health in Residents of Care Homes with Nursing (BHiRCH-NH) study in the UK was a pilot cluster randomised controlled trial of an intervention that was adapted from the INTERACT study, with the addition of co-design involving residents, family carers and nursing home staff to ensure the intervention was applicable to the UK context [6]. Results from the BHiRCH-NH pilot study indicated that over the 6-month follow up period, the number of hospital admissions, ambulances deployed, and ED attendances did not differ significantly between the intervention and control groups [6].

In contrast to these previous studies, the single-site Australian pilot (EDDIE) – the precursor of the multi-site study reported in this paper (EDDIE +) observed a reduction in both annual hospital admissions and average length of stay (19% and 31%, respectively) with a nurse-led intervention focused on hospital avoidance and identifying deterioration in one RAC home [7]. Further, the cost-effectiveness analysis indicated a reduction in cost (-$249,000AUD in the modelled cohort of n = 96) when EDDIE was compared to standard care and a modest improvement of a 0.06 gain in quality-adjusted life-years [7].

### EDDIE + intervention

With promising results from the single site EDDIE pilot [7], the development and scale up from pilot study to multi-site trial was warranted. The development of the EDDIE + intervention [8] and the hybrid effectiveness-implementation stepped wedge randomised controlled trial to evaluate EDDIE + have previously been described [9–11]. As per the pilot study, the EDDIE + intervention comprised four key components: education and training, decision support tools diagnostic equipment, and implementation facilitation (Table A1, A2 – Additional File and https://www.aushsi.org.au/research/projects/eddie-resources/) and was delivered in 11 RAC homes located in regional and metropolitan areas across Queensland, Australia [10]. Strategies were determined by a cross-disciplinary Intervention Working Group with details reported elsewhere [8].

### EDDIE + implementation process

Both the main trial and the implementation process for EDDIE + were theoretically informed by the i-PARIHS framework [12] which proposes that successful implementation results from facilitation of an innovation, in this case EDDIE +, with the intended recipients (RAC staff, residents, and families) in their local, organisational and system context i.e., the individual RAC home, aged care provider and health and aged care system, respectively. The underlying intervention logic [9] was that ongoing facilitation of the multi-component EDDIE + intervention would provide both the skills and the tools for RAC staff to identify early signs of deterioration, communicate collaboratively, and intervene where possible to manage the deterioration and avoid a hospital transfer, resulting in improved resident and family experience, improved workforce satisfaction, and a reduced burden on the hospital and healthcare system.

### Main trial findings

The primary outcome of EDDIE + was the number of hospital bed days accrued by RAC residents [10]. Other effectiveness outcomes included emergency department transfer rates, hospital admission rates, length of hospital stay and discharge outcomes [10]. No significant improvements in these outcome measures were observed post-intervention [11]. The most documented reason for ED transfer during the intervention period was falls (46%) indicating a fundamental misalignment between the intervention and the external policy content around falls transfers, that likely impacted on intervention effectiveness [11].

### Process evaluation

As outlined in the published protocol paper [9], the planned process evaluation aimed to provide more detail on the trial findings, in this case why the intervention did not have the intended effect on the primary and secondary outcomes. Implementation outcomes of interest in the process evaluation included fidelity, acceptability, mechanisms of impact (how the expected changes were hypothesised to occur) and feasibility (assessed through reported barriers and enablers of implementation).

## Methods

Aligned with the theoretically informed implementation process, the process evaluation was guided by the i-PARIHS framework using a mixed methods approach [9]. Data across the 11 homes were collected using a combination of documentary evidence sources (home level context-mapping, training records, field notes and check-in forms), pre and post-intervention validated staff self-efficacy surveys [13], and semi-structured interviews (conducted by female qualitative researchers ELB, PhD; GH, PhD; MA, PhD) with a range of stakeholders including family members, RAC staff (registered nurses, enrolled nurses, personal care workers, residential managers, clinical managers, and other related roles) and internal clinical facilitators.

Additionally, interviews were conducted with the study clinical nurse educator (who provided initial interactive training to nurses and personal care workers), and the study implementation facilitator, (who supported the local internal facilitator who had a responsibility to provide ongoing EDDIE + education), to explore individual home level differences in implementation processes (Tables 1 and 2). Further detail on data collection and methods is outlined in the published process evaluation protocol [9]. Interviewees were not known to researchers prior to study commencement, apart from the study clinical nurse educator and study implementation facilitator who had a role in the study.

**Table 1:** Overview of process evaluation components, data sources and method of analysis [9]

Process evaluation component	Data source/s	Method
Fidelity	Baseline context mapping, activity tracking, check in forms, education sessions delivered	Quantitative
Acceptability	Semi-structured interviews, self-efficacy surveys	Qualitative
Mechanism/s of impact	Self-efficacy surveys, semi-structured interviews	Mixed
Barriers and enablers & Facilitation	Communication and activity tracking, field nots, check-in forms, semi-structured interviews	Qualitative

**Table 2:** Overview of data sources and collection timing

Data Source	Description	Data Collection
Communication and Activity Tracking	Records of EDDIE + nurse educator training, details of home structure, staff numbers. field notes, site contacts, other relevant study updates or interactions with home including staff changes and key personnel	Collected routinely across the trial – updated as needed
Context mapping	Description of home characteristics before EDDIE + intervention i.e., size of home, bed numbers and occupancy, training facilitates, staff structure (residents: nurses), backfill, local primary care providers, other services and local policies and procedures and external hospital avoidance programs	Baseline only
Check In Forms	Tracked number and type of education sessions, hours undertaking role, consumables, staff changes, outbreaks, reflections, successes and priorities	Fortnightly across the trial
Semi-structured interviews	Interviews with RAC staff, residents and family members, EDDIE + LCF and external stakeholders to understand views and experiences with EDDIE +	Close to intervention end
Self-efficacy surveys	Examines both job and team-related self-efficacy with a range of statements on a Likert scale from strongly agree to strongly disagree	Pre and post intervention
Family member or nominated advocate questionnaire	Traffic light system with three questions related to EDDIE + including experience, impact and introduction into other RAC	Collected once – not analysed in this study

Fidelity refers to the extent to which the innovation was delivered as planned. Data to evaluate fidelity of implementation were primarily identified from check in forms and training records to track education delivery and participation, and internal clinical facilitator activity (Table 1). Whilst not a quantitative measure per se, fidelity was assessed as a combination of higher number of activities delivered, relative number of staff involved in education or other activities, a percentage of check-in forms returned, known absence of a designated clinical facilitator, and qualitative information from check-in forms and field notes on use or observed use of equipment and decision support tools. These data were triangulated and used to indicate which sites had more comprehensive delivery of the core components as intended.

Data related to acceptability were extracted from semi-structured interviews and open-ended questions from the staff self-efficacy survey (Tables 1 and 2). Interviews were conducted either over the phone and digitally recorded or using Microsoft Teams (Microsoft Office) with live audio transcription. Interview participants were recruited by the study educator and or implementation facilitator through convenience sampling using a face-to-face approach during study sessions at each RAC home. Participants provided written consent but were not provided with a copy of their transcript for comment or correction. Only the interviewer and interviewee were present during interviews and no repeat interviews were conducted. No participants refused to participate or dropped out, however, some participants did not consent to be recorded, therefore notes were scribed during the interview. For interview transcripts that were audio and visually recorded, transcripts were de-identified and coded in NVivo Qualitative Analysis software (Release 1.6, Version 14, QSR International). Resident interviews were inductively analysed to identify common themes [14] while the staff and RAC stakeholder interviews were mapped to the i-PARIHS framework (Table 1). One author coded the data from qualitative interviews, surveys, and other project documentation. Once the initial analysis was complete, two authors discussed coding to check agreement, where no conflicts arose. A decision to cease interviews was made for pragmatic reasons following significant recruitment challenges.

Self-efficacy data from pre- and post-surveys were entered into SPSS statistical software (Version 2.0.0.0, IBM). Descriptive statistics including gender, age, aged care experience, staff role and facility were presented as mean ± standard deviation (SD) or as frequencies with percentages (%).

Linear mixed effect models were fitted to quantify the association between intervention exposure and self-efficacy scores. Models were fitted separately to total job-related self-efficacy and team-related self-efficacy as the response variables and used responses from participants who completed both pre- and post-surveys. Intervention exposure was included as a categorical covariate (pre- or post-intervention at the date of questionnaire completion). Age, gender, aged care experience (in years), and job role (registered nurse, enrolled nurse, personal care workers, other workers) were included in models as potential respondent confounders. The models included a random intercept for each RAC home to account for correlated responses from participants that worked at the same home at the time of survey. Model results were summarized as regression estimates with 95% confidence intervals and p-values from two-sided Wald testing. The interpretation of hypothesis tests assumed a statistical significance level of 0.05.

## Results

### Description of enrolled homes

11 RAC homes across Queensland participated in the EDDIE + trial with intervention exposure time ranging from 21–239 days during the intervention period (May 2021 to March 2022) (Table 3). Participating RAC homes had different staffing ratios, resident/bed numbers and had varied access to General Practitioner support and local hospital-led avoidance programs.

**Table 3:** Summary of EDDIE + homes with regards to intervention length and staffing

Home	1	2	3	4	5	6	7	8	9	10	11
**Intervention Timing**											
Intervention Exposure	17/5/21	14/06/21	12/07/21	09/08/21	20/09/21	04/10/21	01/11/21	29/11/21	27/12/21	21/02/22	21/03/22
Intervention Days	329	301	273	245	203	189	161	133	105	49	21
**Home structure and staffing**											
Bed numbers*	164	107	140	120	91	100	110	126	101	101	91
Occupancy*	134	100	113	102	78	88	103	114	87	87	83
PCW*	60	70	74	66	59	59	74	72	53	44	60
RN*	18	18	21	21	20	13	13	17	12	9	12
EN*	5	1	4	7	5	4	5	2	2	4	3
GPs*	3	6	3	3	3	4	29	-	2	3	8

PCW = Personal Care Workers; RN = Registered Nurse; EN = Enrolled Nurse; GPs = General Practitioners

^*^Values provided based on initial context mapping, however, were subject to change during the EDDIE + intervention

### Description of interviewees

A total of 20 semi-structured interviews were completed during May to September 2022 with key staff and stakeholders across the 11 homes (Table 4). Interviews ranged from 4 to 24 min. Due to staff turnover during the study period, and increased workload during the pandemic impacting time to either participate in an interview or facilitate connection to other staff and stakeholders, the proposed number of interviews (n = 90) and data saturation was not met despite a prolonged recruitment period. Recruitment of family members and residents also proved challenging under the circumstances (uncertainty and difficulties due to COVID-19), resulting in fewer interviews than anticipated despite repeated attempts to contact potential interviewees.

**Table 4:** Number of EDDIE + interviews by stakeholder group

Stakeholder Group	Number of Interviews
Resident and or family	4
Personal Care Workers	2
Clinical Facilitators	3
Clinical Managers	3
Residential Managers	5
Other*	3
QUT research project staff	2
Total	20

^*^Other refers to those in non-clinical or managerial roles in the RAC home

### Demographics of self-efficacy questionnaire respondents

There were 767 staff who completed the pre-intervention self-efficacy questionnaire and 259 staff who completed the post-intervention questionnaire, 105 of which were matched pairs. Matched pair participant demographics are displayed in Additional File 2, Table A3. In brief, participants were majority female (87%), aged 43.2 ± 14 years and at post intervention had an average experience of 10.4 ± 8.6 years working in aged care. Most respondents were personal care workers (71.4%) or registered nurses (20%).

The following process evaluation results are presented for implementation outcomes related to fidelity, acceptability, mechanism/s of impact, and feasibility (implementation barriers and enablers), reporting on relevant quantitative and qualitative data [15].

### Fidelity

#### EDDIE + training provided and completion of check in forms

Homes received between 14 and 38 training sessions (mean sessions received n = 27) from the study clinical nurse educator. Sessions were scheduled according to staff availability to maximise attendance. The internal local clinical facilitators (LCF) provided between 23 to 263 additional training sessions to personal care workers and between two to 67 sessions to registered and enrolled nurses. Neither the number of sessions delivered by both the clinical nurse educator and LCF or the number of completed check in forms correlated with the time spent in the intervention phase (Tables 3 and 5).

**Table 5:** Fidelity components of EDDIE + across 11 homes

Fidelity Component/Home	1	2	3	4	5	6	7	8	9	10	11
**EDDIE + Training sessions (External Clinical Nurse Educator)**	30	26	35	38	32	25	25	14	26	25	19
Number of CF*	1	1	1,2	1,2	1,2	2	1	1	2	3,1	2
**EDDIE + Training sessions (Internal CF)**											
PCW	109	263	54	23	112	20	17	141	0	0	40
RN/EN	37	67	20	0	46	2	15	37	0	0	8
**CF Check in forms completed n (%)**	12 (52)	20 (83)	12 (55)	4 (22)	16 (100)	9 (53)	14 (93)	12 (92)	2 (18)	2 (33)	3 (60)

PCW = Personal Care Workers; RN = Registered Nurse; EN = Enrolled Nurse; QUT = Queensland University of Technology; CF = Clinical facilitator;

^*^Subject to change throughout the EDDIE + intervention due to staff turnover. CF role could be a single role by one person (1) or job-sharing i.e., dual role (2) or trio role (3)

Only one LCF (Home 5) returned 100% of the check in forms, intended to be filled out on a fortnightly basis during the trial to document EDDIE + activities and training, equipment and resources used, relevant barriers or issues, and successes (Table 5). The remaining homes returned between 18 to 93% of their requested check in forms.

Most homes experienced a vacancy in the LCF role at some point, which affected the delivery of EDDIE +. Despite homes having variable resident numbers, most had one LCF delivering EDDIE + activities, although some had two or three LCFs sharing the role (Table 5).

Overall fidelity to implementation of EDDIE + was mixed and variable across homes. Of the four core components: education and training, decision support tools, diagnostic equipment, and implementation facilitation, we were able to assess the fidelity to education and training and implementation facilitation, with limited ability to determine the use of decision support tools and diagnostic equipment as this was not routinely measured.

### Acceptability

To determine the acceptability of the EDDIE + intervention, qualitative data were collected from open-ended responses to the self-efficacy surveys (RAC staff only) that were administered pre- and post-intervention, and semi-structured interviews (RAC staff, project-related staff, and family members).

Quotes from self-efficacy surveys were coded in the format [RN1201_6], whereas data from semi-structured interviews were coded [Family_1_Home_6] or [CM_3_Home_6]. Supplementary quotes are available in Additional File, Table A6.

#### Family perspective

Interviews with family members indicated that they had limited awareness of the EDDIE + intervention but generally supported the idea of minimising hospital transfers where possible. This was seen to benefit both the residents of the home and the wider health system. Family members described how disruptive a hospital admission could be to a loved one, especially for those with dementia or cognitive impairment.

“Often, it’s not necessary for her to go to hospital and it’s better if she doesn’t have to”. [Family_1_Home_10]

“Going to the hospital is very stressful for her just it’s unfamiliar, a bit frightening and yeah just generally disruptive for her”. [Family_2_Home_6]

Family members also highlighted the impact of hospital admissions on the health care system and how sending residents to hospital may warrant ‘unnecessary’ costs and potentially invasive tests.

“Mum had a situation last August where she had to go to hospital for a couple of days and the ambulance had to park up the side street and wheel her down past all the houses to get to the front of the hospital. So ambulance ramping is huge. Yeah, I certainly wanna keep people out of our hospitals as much as possible.” [Family_1_Home_6]

#### Staff and stakeholder perspectives

Feedback from staff and stakeholders regarding the EDDIE + intervention was overwhelmingly positive. Interviewees described the intervention as ‘brilliant’ and highlighted the need for education and upskilling in the RAC sector, especially with the inclusion of personal care workers.

“So, I think the actual program itself is brilliant, I think it was really great”….We’re a lot better for having EDDIE+ then for not having it.” [RM_1_Home_1]

“I've been working in aged care for so many years and sometimes some things change. But I don't recognize, that's a deterioration so it's sort of this program reminds me.”[PCW_1_Home_6]

The ability to upskill and be provided with education relevant to clinical practice was appreciated by RAC staff and their managers.

“I think everyone here, we’re all for that sort of intervention to increase people's capability. And it goes beyond that, it's an important point to recognize that it goes beyond just improving the care …. [it] actually improves peoples job satisfaction” [Other_3]

### Mechanism of impact – self-efficacy

The study logic model hypothesised changes in staff self-efficacy as the key mechanism for improving outcomes, resulting from increased staff knowledge, skills, and competence, and improved team communication and functioning.

#### Effect of EDDIE + on job and team related self-efficacy

Mixed model analyses indicated that the intervention did not have a strong effect on either job-related self-efficacy scores (Estimate = 0.36, 95% CI = –0.52 to 1.26, p = 0.43), or team-related self-efficacy scores (Estimate = –0.57, 95% CI = –1.74 to 0.58, p = 0.34) (Additional File 2, Tables A4 and A5).

The self-efficacy questionnaire also included a qualitative response option with 3 open-ended questions: What was the best part of EDDIE +? (n = 219 responses); what would have made EDDIE + better? (n = 169); and any other comments about EDDIE +? (n = 132). Qualitative analysis identified themes that provided support for the hypothesised improvements in staff knowledge, skills, confidence and communication.

#### Increased knowledge and skills

EDDIE + was perceived by staff as increasing their knowledge, skills, and understanding of how to identify and manage early signs of resident deterioration.

“The best part of the EDDIE+ program is gaining knowledge about deterioration of the resident which will help to save them from several things such as falls, UTI and so on” [PCW1222_12]

Access to new equipment such as the bladder scanner and ECG machine was seen to reduce the need to call for an ambulance and enabled RAC staff to better communicate the resident’s status with the additional information from the new equipment. This was seen as an advantage not only for the health system, but for the resident’s comfort as well.

“We don't really have a doctor residing or like regular doctor visiting every day. So there's a little bit of difficulty contacting doctor and follow up on resident’s condition. We got the vital signs monitor and bladder scanner, so it was more helpful to follow up on. We were using the equipment before we contact the doctor, so we were like able to provide all the information in one go”. [CF_4_Home_7].

#### Increased confidence and competence

EDDIE + training was perceived by some staff as increasing their confidence. Staff noted that it was the “*best training to make yourself confident*” [PCW1176_8] and to *“gain knowledge and confidence”* [PCW1204_7]. Some staff reported feeling enhanced confidence in their own competency at work, including using the equipment:

“It gave me confidence and additional knowledge. I am proud to say I was able to perform it (using the bladder scanner) during one of my shifts” [RN1230_12]

Feelings of confidence and competence tied in closely with enhanced knowledge and skills. Equipped with the knowledge acquired during the EDDIE + training, staff reported learning new *“ways to approach problems”* [RN1335_5] and new approaches to caring for older people:

“It has helped to pay more attention to client’s care holistically and identify early signs of

deterioration which allows proper care” [PCW1130_14]

#### Enhancing communication

Communication between nurses and personal care workers (PCWs) was a prominent theme throughout the data. PCWs commented that EDDIE + gave them a *“better way of communication with RNs”* [PCW1340_4] as they were “*taught how to open a dialogue with nurses” [PCW1274_3]* through *“learning new skills and communication techniques”* [PCW1076_7]. Enhanced understanding and communication skills, using the EDDIE + provided tools, gave the PCWs confidence in communicating to nurses about residents when the PCWs noticed signs of deterioration, *“giving clarity and uniformity to communicate with nursing and other staff”* [PCW1316_5]. Many of the PCWs reported feeling empowered by this enhanced communication, that they now had a voice and could speak up for those in their care.

“After I introduced all the communication tools from EDDIE+, care staff were more motivated and like encouraged to report it because they're happy to see what change is made and what like they noted and made the difference in residents’ condition.” [CF_4_Home_7]

The skills and training with specialised equipment also fostered a more collaborative environment between RAC staff, GPs, and other service providers. Having access to data from the equipment resulted in better communication and cooperative efforts to work towards the common goal of the residents’ health.

“A home is not a hospital, and it doesn't have medical care available. To me it's super important, if you can get on to a GP, then the premise behind it is the nurse has got accurate information to give the GP to determine an outcome. …. If you can't even articulate what's wrong, that's not helpful. And also, if the GP is not available to take call once again when the ambulance arrives, when you're calling the ambulance, you've got real information. Those things are extremely valuable.” [Other_2]

In summary, although no statistically significant differences in self-efficacy were apparent pre- and post-implementation, the qualitative data from both the semi-structured interviews and self-efficacy surveys suggested improvements in knowledge, skills, confidence, competence and communication for both nursing staff and PCWs.

### Feasibility: identified barriers and enablers

#### Barriers

Contextual barriers were prominent in the RAC homes during the study, particularly staff shortages and high turnover within aged care, exacerbated by the COVID-19 pandemic and other factors. This necessitated additional infection control measures, including lockdowns and staff working within ‘bubbles’ with limited flexibility to move, for example, between floors or areas of the home. Increased workload and pressures on staff sometimes led to a situation where hospital transfer of a resident was inevitable.

“I actually had one of my nurses rang me late last night they wanted to send someone to hospital and I’m trying to work out why and if there’s anything we can do and you can just hear the phone in the background going mad, we’re short staffed and there’s people yelling and stuff and it’s just like nah call QAS (Queensland Ambulance) and get them out because you don’t have the resources right now”. [RM_4_Home_11]

Workload and staffing issues also impacted the feasibility of the internal facilitator role, both in terms of filling the role and enacting the expected responsibilities of the role. For example, although project funding was available for RAC homes to backfill the internal facilitator role one day a week, this was not always possible due to staff shortages and the need to cover essential work.

“I do really like the program. I'm just disappointed that it wasn't done as well as I could have done it just with all the different roadblocks …..I didn't find it manageable, but it's not because of what was expected of me it's because like I was constantly getting pulled away from doing it.” [CF_2_Home_10]

High levels of staff turnover without consistent levels of local facilitation to support the ongoing training and engagement created barriers to embedding EDDIE + into routine practice.

“It’s staff availability, it doesn’t matter how you, how you dress it up, if the staff are unable to attend the session or we have the turnover and lack of retention of staff it’s a problem. In some cases, we have 30% turnover of staff only from a clinical team between each quarter. So you may educate people on this particular equipment and identifying deterioration but then they leave and you get a new person in so yeah it’s maintaining the consistency”. [Other_3]

Alongside the practical difficulties of a lack of time to engage in EDDIE + training, some concerns were also raised about the culture of aged care and the ability to apply learning in practice.

“The culture in aged care that they’ve all been I guess mentored into where you’re task focused and you’ve got a lot of work to do in a single shift and you put your head down and do it. So, there’s not a lot of reflection of practice and there’s not a lot of thought about the global picture which is what you need when you’re looking at deterioration. So, it’s a lot around culture but it’s a lot around lack of skill and experience and inability to take this information and reflect on it and add it to your practice.” [Other_1]

An additional policy-related barrier identified was around the management of residents who fall, which could lead to a hospital transfer regardless of the severity of the fall.

“(Name of Home) policy is anyone with anticoagulants you have to send them to hospital and with unwitnessed falls”. [RM_5_Site_6]

“Our hospitalisation rate when I checked the data, it actually increased but that increase is the mainly due to the falls…We have two frequent fallers, then if they're falling almost every day, the fall rate goes really high”. [RM_5_Site_6]

#### Enablers

Whilst contextual factors presented major barriers during implementation, interviewees also identified several enablers of EDDIE + implementation. These included the EDDIE + clinical nurse educator and implementation facilitator, managers who were supportive of the EDDIE + intervention, availability of infrastructure (e.g., access to information and technology) and the improved communication skills that developed through participation in EDDIE +.

Overall, staff were committed to sustain the learnings from EDDIE + and felt that it should continue in the long term, including incorporating EDDIE + components into policies or guidelines to enable the early identification of deterioration and including the EDDIE + education modules in the organisation’s learning management system for future use.

A strong focus on the resident was also identified as an enabler with person-centred care at the forefront of minds and a potential motivator for RAC staff.

“They're (RAC staff) happy to see what change is made and if they made the difference in residents condition” [CF_4_Site_7]

Interviewees also commented on the importance of the facilitation and mentorship that EDDIE + provided which could help to bridge the gap for novice practitioners coming into the aged care workforce and improve skills and confidence.

“I've recruited graduate nurses for decades and a graduate goes into a clinical environment with a novice skill set. But you know where most of the learning occurs, it's actually from the nurses around you that are experienced, right? You learn something, they show you something. It's that mentorship and job experience.” [Other_2]

At a home level, having the right person with the right skills in the facilitator role acted as an enabler, as did the input and support from the external clinical nurse educator and implementation facilitator.

“As soon as we said that she was going to be the facilitator everyone's eyes lit up, she was funny and animated and really good” [QUT_project_team_1]

“I think the guys who did the education were very approachable and that made it easier just with their approach and everything made it easier on the staff and that worked really well” [RM_2_Home_7]

#### Integration of results

**Fig. 1:**
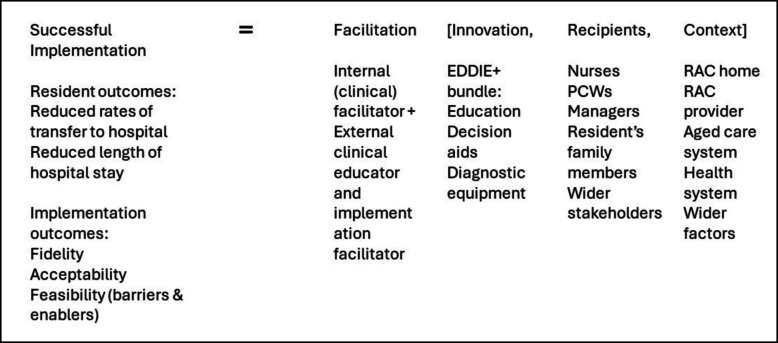
Applying i-PARIHS to the EDDIE + intervention

When considering the original logic model that hypothesised successful implementation would be achieved through facilitation of the EDDIE + intervention to staff members in the RAC home (Fig. 1), the process evaluation results indicate that this did not occur as planned.

**Fig. 2:**
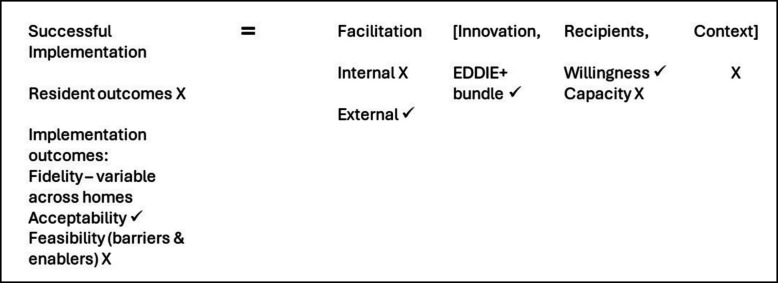
Integration of EDDIE + process evaluation results in relation to the i-PARIHS framework

Successful implementation was limited by internal facilitation, recipient capacity, contextual issues in the home and feasibility (Fig. 2). Despite this, acceptability, and willingness of staff to engage with EDDIE + was high.

## Discussion

This embedded process evaluation considered the implementation of the EDDIE + intervention across 11 RAC homes in Queensland, Australia. Given the null main trial outcome [11], we were particularly interested to understand why the EDDIE + intervention did not work as expected in addressing this question, we considered a range of implementation factors, including acceptability, fidelity and feasibility.

The EDDIE + intervention encompassed a bundle of different components, including embedding recognised implementation strategies such as education and training and facilitation [16]. The education and training component included both fixed i.e., initial face to face training with external facilitator with a suite of topics related to deterioration and response, and flexible components i.e., length, intensity and delivery methods of training [8]. However, whether the content of the education aligned well with the actual reasons for resident transfer to hospital is questionable. Based on the previous EDDIE pilot study, the educational resources targeted eight aspects of deterioration that could lead to potentially avoidable hospitalisation [8]. However, of these eight areas, falls, accounted for 46% of all admissions during the EDDIE + intervention period, whilst the other seven combined (for example, constipation, dehydrations, dyspnoea) accounted for just 17% of hospital transfers [11]. Furthermore, pre-existing policies necessitated hospital transfer, for example, for an unwitnessed fall, meaning that hospital transfers might not be ‘avoidable’ in the case of a resident falling. In turn, this has implications for the design and implementation of RAC-focused hospital avoidance programs and highlights the prevalence of the ‘falls paradox’ as a driver of unavoidable hospitalisations. Future policy changes may address this identified paradox. This finding also highlights the need for careful planning and consideration when scaling up pilot and single site interventions, as this was not identified as an issue or barrier in the single-site EDDIE study.

The strategy for implementing EDDIE + was theoretically informed by the i-PARIHS framework, with facilitation as the overarching strategy to address identified determinants of implementation [12, 17]. Thus, the study employed a model of internal–external facilitation and undertook pre-implementation context mapping at the individual RAC home level to identify potential barriers and enablers related to the i-PARIHS constructs of innovation, recipients and context [8, 10]. The facilitation approach was then tailored to address the identified barriers and enablers, for example, by having core and adaptable components, such as flexible methods of implementing the training program to build staff knowledge and skills and ‘quarantined’ time dedicated to facilitation (i.e., 0.1 to 0.2 FTE or 1–2 days per week) [8]. However, the process evaluation findings suggest that the theoretical proposition underpinned by i-PARIHS did not function as planned to achieve ‘successful implementation’, which had previously been observed in the pilot study [7]. The reasons for this are multiple and highly context-dependent.

With regards to the innovation factor, there was an overall high level of support from staff, managers, family members and other stakeholders. Most interviewees felt that the principles behind EDDIE + aligned with their beliefs about keeping residents out of hospital where possible and appropriate, indicating support for and acceptability of the intervention. However, contextual barriers such as staff turnover, and high workload were substantial and greatly impacted fidelity of implementation, including the delivery of educational sessions, which varied considerably across homes. The clinical facilitator role, a key component of the EDDIE + intervention was often vacant (or unfilled) due to staffing and workload pressures and limited capacity to source staff to provide backfill. As a result, the ongoing delivery of education and training to staff did not occur as planned via the embedded local facilitation model. Similarly, the wider aspects of the clinical facilitator role, including communication, collaboration and support to deliver the multi-component EDDIE + program were affected, in turn impacting the degree to which expected changes were socialised into routine practice. The inter-site variability in fidelity that occurred within the context of universal pressures including COVID-19 and staffing, that all homes experienced, may also be reflective of other factors related to the innovation itself, organisational culture and leadership style, or even factors within the broader context such as geography. While most interviewees reported the EDDIE + intervention had relative advantage and a degree of fit within the context, it is possible that with our limited number of interviews, we may not have captured alternative views and beliefs around the intervention where participants may have had opposing views. Further, variation in experience of clinical managers and manager turnover as well as the ‘task-focused’ mindset and culture in aged care may have impacted fidelity with a clear need for ongoing coaching, mentoring and upskilling opportunities.

Issues relating to staff resources, competing demands and instability of nursing home leadership were similarly reported in the US INTERACT [5] study of 85 nursing homes which found no change in ED admissions or hospital transfers following implementation of a training and education program [5]. However, unlike EDDIE +, participants in the INTERACT study reported varying degrees of beliefs and motivation around reducing hospitalisations and the benefits for residents as well as concerns around managing residents within the home from a legal and regulatory perspective [5].

From a nursing and PCW perspective, despite their acceptance of EDDIE + and identified benefits from being involved in the project, their capacity to implement was severely impacted by workload and staffing pressures—pressures that were exacerbated by wider contextual factors related to the COVID-19 pandemic and environmental events. Together, this set of contextual barriers severely constrained the ability to enact facilitation in the way intended within the i-PARIHS framing of the study, where facilitation was positioned as the ‘active ingredient’ of implementation. The internal clinical facilitator roles were particularly affected by contextual challenges, leading to turnover of identified facilitators, difficulty making time to undertake the role as planned, despite the availability of funding for staff backfill, and inconsistency in how the role was applied across the 11 homes.

The education and/decision support tools provided as part of the intervention were well received and had a qualitative impact on perceived self-efficacy. This led to staff feeling empowered to recognise changes in a resident’s condition and escalate when needed using the communication tools provided, although no quantitative effect on job or team-related self-efficacy was observed. However, the small number of matched pairs (n = 105) due to the drop-off in survey responses limits our quantitative findings.

These findings raise several discussion points related to the use of facilitation as an implementation strategy and the current theoretical framing of facilitation, as proposed by i-PARIHS [12, 17, 18]. In terms of the implementation strategies employed, the focus was on facilitation and using the external-internal facilitation approach as a capacity building model to deliver ongoing education and support around clinical deterioration to nurses and personal care workers. Whilst these are implementation strategies with some supporting evidence [19], it could be that adding in other evidence-informed implementation strategies, such as audit and feedback of patient transfer data, would have further strengthened the implementation process. However, the practicality of incorporating additional strategies would have proved difficult in the context of the challenging circumstances faced by the RAC homes.

In turn, this raises questions about the facilitation-context relationship. Whilst i-PARIHS suggests that facilitation can actively address contextual barriers, this was not the case in the EDDIE + study, where barriers relating to the pandemic, staff turnover and shortages and natural disasters were not modifiable. Other implementation studies have demonstrated similar issues where facilitation interventions have produced a null effect, typically in the face of significant local, organisational and system-level contextual barriers [20, 21]. This could be due to one of a number of factors, such as selecting, recruiting and preparing individuals to take on the facilitator role, the level of support and mentorship available, and/or the intensity or ‘dose’ (frequency and duration) of facilitation provided [20, 22]. Equally, there could be a point at which contextual barriers are too overwhelming and no amount of internal or external facilitation is able to overcome them without addressing higher level organisational and system barriers. This has implications for the i-PARIHS determinants as currently described and is an important area for research in the future.

### Limitations

As previously acknowledged in the process evaluation protocol paper, EDDIE + was implemented with only one aged care provider, operating multiple facilities across Queensland. Though the homes included regional and metropolitan sites, our findings may not be directly transferable to other aged care providers across Australia. However, despite the COVID-19 pandemic, lockdowns, other virus outbreaks, and floods that impacted Queensland during implementation, the EDDIE + study was still completed. These challenges resulted in evolving changes to staffing structures, i.e., limiting contact with other staff by forming work area ‘bubbles’, as well as a high turnover of staff (RNs, PCWs, CFs, and those in managerial positions) all of which impeded the ability to deliver EDDIE + as intended and maintain a consistent internal clinical facilitator role across homes. Facilitation was considered a key ingredient in the implementation of the EDDIE + intervention; however, limited data was collected on the functionality of the role due to role vacancies and a limited number of interviews with clinical facilitators and RAC home staff. As a result, we were unable to robustly examine the role of the facilitator as intended based on our original process evaluation protocol paper. Additionally, the EDDIE + bundle included decision support and communication tools and new diagnostic equipment [10] and we were unable to comprehensively evaluate factors relating to the adoption of these components of the intervention.

Due to the COVID-19 pandemic and high rate of staff turnover, there was a considerable drop off between pre- and post-survey responses, with only 105 matched pairs, limiting inferences made from the quantitative analyses. Further, we did not achieve our intended sample size for semi-structured interviews (n = 20/90). Despite multiple follow-ups, the RAC staffing crisis negatively impacted our ability to recruit staff for interviews. Further, recruiting family members and residents was challenging, resulting in limited data from this perspective, despite being a key stakeholder group. Additionally, as interviews ranged in length considerably (4 −24 min), we were unable to reach data saturation, therefore limiting the depth of our qualitative analysis.

Overall, this process evaluation collected process data related to acceptability, self-efficacy, and fidelity, but reports limited data related to sustainability and other process outcomes which may have further strengthened our ability to draw conclusions based on the results.

### Implications and future recommendations

This process evaluation provides important considerations for implementing interventions aimed at the early detection and management of deterioration in residents of aged care homes. The findings suggest that RAC staff, other stakeholders and family members support the premise of EDDIE +. However, without sufficient staffing levels, interventions that operate with internal facilitators may have limited success in the current aged care workforce and staffing context.

Within the study sites, the qualitative interviews indicated a preference for the continuation of EDDIE + principles or an iteration of the training and resources that could be delivered on an ongoing basis, either online, or by an external facilitator/dedicated and independently resourced supernumerary facilitator. Post-pandemic interventions should continue to dedicate time to forge stronger links with GPs and external hospital-led avoidance programs, particularly in relation to accessing out-of-hospital medical support, and to advocate for shifting some lower acuity services e.g. x-ray either into, or closer to RAC homes to enable this care to be done in-situ or out of hours. Further, capturing these subtle ‘intermediate’ outcomes such as how often increased GP communication, or use of diagnostic equipment impacted resident care plans, may assist in bridging the gap between process and effectiveness outcomes to reveal subtle practice change not typically captured in intervention trials.

Additionally, novel methods of incorporating the resident and family member voice may need to be developed to capture this important stakeholder group in process evaluations and effectiveness analyses.

## Conclusion

The process evaluation of EDDIE + demonstrates that despite a high level of acceptability of the EDDIE + intervention, challenges related to fidelity and feasibility were variable across homes and contextual factors presented significant barriers to implementation that may explain the lack of observed effect on hospitalisation-related outcomes. The findings highlight the complexity of changing practice within challenging contexts such as the RAC environment and unpredictable situations such as global pandemics. This highlights the need for careful attention to both intervention and implementation design and conduct.

## Supplementary Information

Additional file 1.

Additional file 2.

Additional file 3.

## Data Availability

The data is available from the corresponding author upon reasonable request.

## Abbreviations

ANZCTR: Australia New Zealand Clinical Trial Registry
CF: Clinical facilitator (internal facilitator within the RAC home)
CM: Care Manager
EDDIE: Early Detection of Deterioration in Elderly Residents (single home pilot)
EDDIE +: Early Detection of Deterioration in Elderly Residents (multi-home intervention)
EN: Enrolled Nurse
GEDI: Geriatric Emergency Department Intervention program to help frail older people access care (Sunshine Coast and HHS)
GP: General Practitioner
HREC: Human Research Ethics Committee
i-PARIHS: Integrated Promoting Action on Research Implementation in Health Services
PCW: Personal Care Worker
RAC: Residential Aged Care
RN: Registered Nurse
QUT: Queensland University of Technology

## References

[1] Australian Medical Association. Putting health back into aged care. Barton, ACT; 2021.

[2] Arendts G, Howard K. The interface between residential aged care and the emergency department: a systematic review. Age Ageing. 2010;39(3):306–12. 20176712 10.1093/ageing/afq008

[3] Boockvar KS, Gruber-Baldini AL, Burton L, Zimmerman S, May C, Magaziner J. Outcomes of infection in nursing home residents with and without early hospital transfer. J Am Geriatr Soc. 2005;53(4):590–6. 15817003 10.1111/j.1532-5415.2005.53205.x

[4] Caplan GA, Ward JA, Brennan NJ, Coconis J, Board N, Brown A. Hospital in the home: a randomised controlled trial. Med J Aust. 1999;170(4):156–60. 10078179

[5] Kane RL, Huckfeldt P, Tappen R, Engstrom G, Rojido C, Newman D, et al. Effects of an Intervention to Reduce Hospitalizations From Nursing Homes: A Randomized Implementation Trial of the INTERACT Program. JAMA Intern Med. 2017;177(9):1257–64. 28672291 10.1001/jamainternmed.2017.2657 PMC5710568

[6] Elizabeth LS, Alexandra F, Alan B, Katherine F, Rachael H, Louise M, et al. Pilot cluster randomised trial of an evidence-based intervention to reduce avoidable hospital admissions in nursing home residents (Better Health in Residents of Care Homes with Nursing—BHiRCH-NH Study). BMJ Open. 2020;10(12):e040732. 10.1136/bmjopen-2020-040732 PMC7737107 33318118

[7] Carter HE, Lee XJ, Dwyer T, O’Neill B, Jeffrey D, Doran CM, et al. The effectiveness and cost effectiveness of a hospital avoidance program in a residential aged care facility: a prospective cohort study and modelled decision analysis. BMC Geriatr. 2020;20(1):527. 33287716 10.1186/s12877-020-01904-1 PMC7720399

[8] Allen MJ, Carter HE, Cyarto E, Meyer C, Dwyer T, Oprescu F, et al. From pilot to a multi-site trial: refining the early detection of deterioration in elderly residents (EDDIE +) intervention. BMC Geriatr. 2023;23(1):811. 38057722 10.1186/s12877-023-04491-z PMC10698876

[9] Bracci E, Allen M, Carter EH, Cyarto L, Dwyer T, Graves N, et al. Protocol for a process evaluation of a stepped wedge randomised controlled trial to reduce unnecessary hospitalisations of older people from residential aged care: the EDDIE+ study. BMJ Open. 2023;13(2):e066857. 36797014 10.1136/bmjopen-2022-066857 PMC9936275

[10] Carter HE, Lee XJ, Farrington A, Shield C, Graves N, Cyarto EV, et al. A stepped-wedge randomised controlled trial assessing the implementation, effectiveness and cost-consequences of the EDDIE+ hospital avoidance program in 12 residential aged care homes: study protocol. BMC Geriatr. 2021;21(1):347. 34090368 10.1186/s12877-021-02294-8 PMC8179705

[11] White NM, Lee XJ, Allen MJ, Graves N, Harvey G, Shield C, et al. The impact of a multi-component hospital avoidance programme in residential aged care homes: a stepped-wedge cluster randomised trial. Age Ageing. 2025;54(10):afaf275. 41052261 10.1093/ageing/afaf275 PMC12499756

[12] Kitson A, Harvey G, McCormack B. Enabling the implementation of evidence based practice: a conceptual framework. Qual Health Care. 1998;7(3):149. 10185141 10.1136/qshc.7.3.149 PMC2483604

[13] Riggs ML, Warka J, Babasa B, Betancourt R, Hooker S. Development and validation of self-efficacy and outcome expectancy scales for job-related applications. Educ Psychol Meas. 1994;54(3):793–802.

[14] Bengtsson M. How to plan and perform a qualitative study using content analysis. NursingPlus Open. 2016;2:8–14.

[15] Bracci E, Allen M, Carter HE, Cyarto L, Dwyer T, Graves N, et al. Protocol for a process evaluation of a stepped wedge randomised controlled trial to reduce unnecessary hospitalisations of older people from residential aged care: the EDDIE+ study. BMJ Open. 2023;13(2):e066857. 36797014 10.1136/bmjopen-2022-066857 PMC9936275

[16] Powell BJ, Waltz TJ, Chinman MJ, Damschroder LJ, Smith JL, Matthieu MM, et al. A refined compilation of implementation strategies: results from the expert recommendations for implementing change (ERIC) project. Implement Sci. 2015;10(1):21. 25889199 10.1186/s13012-015-0209-1 PMC4328074

[17] Harvey G, Kitson A. Parihs revisited: from heuristic to integrated framework for the successful implementation of knowledge into practice. Implement Sci. 2016;11(1):33. 27013464 10.1186/s13012-016-0398-2 PMC4807546

[18] Harvey G, Kitson A. Implementing evidence-based practice in healthcare: a facilitation guide. Abingdon, Oxon.: Routledge; 2015.

[19] Wilson P, Kislov R. Implementation Science. Cambridge: Cambridge University Press; 2022. Available from: https://www.cambridge.org/core/product/9E9361E2F6C1A3B894C6D202031ECD19.

[20] Rycroft-Malone J, Seers K, Eldh AC, Cox K, Crichton N, Harvey G, et al. A realist process evaluation within the facilitating implementation of research evidence (FIRE) cluster randomised controlled international trial: an exemplar. Implement Sci. 2018;13(1):138. 30442165 10.1186/s13012-018-0811-0 PMC6238283

[21] Bucknall TK, Harvey G, Considine J, Mitchell I, Rycroft-Malone J, Graham ID, et al. Prioritising Responses Of Nurses To deteriorating patient Observations (PRONTO) protocol: testing the effectiveness of a facilitation intervention in a pragmatic, cluster-randomised trial with an embedded process evaluation and cost analysis. Implement Sci. 2017;12(1):85. 28693596 10.1186/s13012-017-0617-5 PMC5504605

[22] Baskerville NB, Liddy C, Hogg W. Systematic review and meta-analysis of practice facilitation within primary care settings. Ann Fam Med. 2012;10(1):63. 22230833 10.1370/afm.1312 PMC3262473

