# Supplementary material for: Implementing an intervention to facilitate early detection of deterioration in aged care residents: process evaluation of the EDDIE + trial
Source: Implement Sci. 2026 Feb 16;21:22. doi: 10.1186/s13012-026-01484-5 (PMC13011326; doi:10.1186/s13012-026-01484-5)
Supplement: Supplementary file 1 — Additional file 1. [file 13012_2026_1484_MOESM1_ESM.docx]

**Additional File 1 – Supplementary Intervention Information**

**Table A1.** Overview of EDDIE+ intervention components as adapted from (1) and (2)

| **EDDIE+ component** | **Examples** |
| --- | --- |
| Education and Training | - Education related to 8 areas of deterioration   - Delirium, constipation, dehydration, dyspnoea, cardiac, falls, urinary tract infections and palliative care - Clinical scenarios and ‘case studies’ for training to support and reinforce ongoing learning |
| Decision Support and communication tools | - ISBAR for nursing staff to aid communication with other clinical teams and or externally i.e., GP or hospital outreach - “Stop and Watch” and “CUS” (Concern, Unsafe, Serious/Safety issue) for internal communication within the RAC home - Clinical parameters and traffic light tool |
| Diagnostic Equipment | - Vital signs monitor inclusive of heart rate, blood pressure and oxygen saturation - Electrocardiogram - Bladder scanners |
| Implementation Facilitation and support | - External facilitation and ongoing implementation facilitation from research team |

**Table A2.** Overview of fixed and flexible elements of EDDIE+ intervention adapted from (3)

| **EDDIE+ COMPONENT 1: Education and training** | | | |
| --- | --- | --- | --- |
| **Elements/key activities** | **Fixed elements** | **Flexible elements (to be determined following initial context mapping)** | **Degree of flexibility (extensive, partial, limited, none)** |
| Initial face-to-face training on early identification of deterioration and response, including roles/responsibilities of each staff member. | Initial training delivered by a nurse educator employed as part of the EDDIE+ study.  Training must be attended by all RNs, ENs and AINs (unless staff are on leave during this period)  Training must cover clinical management practices for specific conditions identified as likely to result in hospitalisation (e.g. UTIs, chest pain, falls, delirium, dehydration, etc).  Training must cover the use of relevant decision support tools and diagnostic equipment.  Training must position EDDIE+ as a research study will a strong evaluation component, and emphasise the focus on generating new evidence to inform decision making. This will include an overview of why the study is needed (i.e. the nature of the problem) and where funding is coming from.  Training must include an element of executive support for the program. | Length, intensity, delivery methods and the depth of content coverage training can be adapted to suit the needs of each site | Partial |
| Educational materials toolkit | A core set of materials will be developed that all sites must cover | Additional materials may be developed to meet needs of each site | Partial |
| Facilitator guide to be developed as resource to support EDDIE+ facilitators in their role. Will include information on the nature of study team support available throughout the project as well as a set of resources to help guide the facilitation (e.g. templates for documenting engagement activities and/or other data collection requirements) | Provided to the EDDIE+ facilitator at each site |  | Fixed |
| **EDDIE+ COMPONENT 2: Decision Support and communication tools** | | | |
| **Elements/key activities** | **Fixed elements** | **Flexible elements** | **Degree of flexibility (extensive, partial, limited, none)** |
| Core decision support tool covering clinical decision-making guidelines for managing deterioration across a number of conditions | All sites must implement a core decision support tool that is introduced in initial training and reinforced at staff meetings | - The number and type of conditions covered by the tool may differ depending on the needs at each site. - The form of the tool (e.g. flip chart, online resource, App-based resource) may differ at each site. | Partial |
| Observation chart (track and trigger tool) as hard copy |  | Optional depending on site preferences/requirements | Extensive |
| Communication tool (e.g. Situation, Background, Assessment, Recommendation) |  | Optional depending on site preferences/requirements | Extensive |
| **EDDIE+ COMPONENT 3: Diagnostic Equipment** | | | |
| **Elements/key activities** | **Fixed elements** | **Flexible elements** | **Degree of flexibility (extensive, partial, limited, none)** |
| Provision of: Bladder scanner, ECG machine, Vital signs monitor, oximeter | Each site will be assessed for their equipment needs and will receive training on equipment as part of the initial face to face training sessions. | The type and amount of equipment will be tailored to meet the needs of each site | Partial |
| **EDDIE+ COMPONENT 4: Implementation facilitation and support** | | | |
| **Elements/key activities** | **Fixed elements** | **Flexible elements** | **Degree of flexibility (extensive, partial, limited, none)** |
| Bolton Clarke EDDIE+ facilitator | Each site will have one internal facilitator who is in a clinical leadership position within the RAC home. The EDDIE+ facilitator will have quarantined time (up to 1 day per week) to dedicate to implementation and study activities including stakeholder engagement, record keeping, liaising with EDDIE+ project staff and mentoring/coaching of other care staff. A ‘facilitator guide’ will be developed as a resource manual to assist facilitators in this role. | Time dedicated to facilitation may vary (between 0.1 to 0.2 FTE)  Specific activities may vary depending on context assessment and implementation strategies that are adopted. | Partial |
| Ongoing executive/management support | RAC home leadership to have a presence at the initial training session to reinforce BC support for the program and confirm their approval for a change in usual practice.  EDDIE+ facilitator to coordinate brief but regular communication with RAC home leadership to act as a continuous feedback loop (e.g. fortnightly project updates, standing item at management meetings, etc) | Nature of communication and feedback to suit the context and needs of each site. | Partial |
| **Clinical support channels**: Access to clinical support from medical personnel e.g. hospital in-reach team (e.g. RASS), clinical lead nurses, nurse practitioner, nurse educator, geriatrician, wound specialist) | All sites must establish channels for RNs/ENs/AINs to effectively communicate any concerns regarding patient deterioration and/or need for hospital transfer | Communication channels will be tailored to site context and needs e.g. RaSS, local GP, etc | Partial |
| **Internal staff support networks:** Ongoing internal clinical support from NP/RAC team, clinical facilitators, local GPs. May include elements of ongoing coaching or mentoring. | All sites must establish internal support networks for staff to feel supported in providing the level of care required of the intervention | Nature and form of support networks/channels may be tailored to each site | Partial |
| Alignment of education and decision support materials with organisational policies and procedures to support long term sustainability | Led by the EDDIE+ facilitators, all sites to work with study team to align decision support materials with organisational policies and practices, and embed these into business as usual. | Site specific policies and procedures may also need to be aligned with study materials | Partial |
| **GP practice engagement:** GPs to be recognised as key decision makers in RAC home hospital transfers. Initial and ongoing engagement required to build the trust of GPs in RAC home clinical management of residents. | GP engagement activities to occur in all sites | Nature of engagement, and number of practices engaged, to suit needs of individual sites. | Partial |
| **Family engagement:** Families to be recognised as key stakeholders with initial and ongoing engagement activities to occur | Family engagement activities to occur in all sites | Nature of engagement to suit needs of individual sites. | Partial |

1. Allen MJ, Carter HE, Cyarto E, Meyer C, Dwyer T, Oprescu F, et al. From pilot to a multi-site trial: refining the Early Detection of Deterioration in Elderly Residents (EDDIE +) intervention. BMC Geriatrics. 2023;23(1):811.

2. Bracci E, Allen M, Carter HE, Cyarto L, Dwyer T, Graves N, et al. Protocol for a process evaluation of a stepped wedge randomised controlled trial to reduce unnecessary hospitalisations of older people from residential aged care: the EDDIE+ study. BMJ Open. 2023;13(2):e066857.

3. White NM, Lee XJ, Allen MJ, Graves N, Harvey G, Shield C, et al. The impact of a multi-component hospital avoidance programme in residential aged care homes: a stepped-wedge cluster randomised trial. Age and ageing. 2025;54(10):afaf275.
