# Supplementary material for: Implementing an intervention to facilitate early detection of deterioration in aged care residents: process evaluation of the EDDIE + trial
Source: Implement Sci. 2026 Feb 16;21:22. doi: 10.1186/s13012-026-01484-5 (PMC13011326; doi:10.1186/s13012-026-01484-5)
Supplement: Supplementary file 2 — Additional file 2. [file 13012_2026_1484_MOESM2_ESM.docx]

**Additional File 2. Supplementary EDDIE+ process evaluation data**

**Table A3.** Matched analysis - Demographics of staff involved in the EDDIE+ intervention who completed both Pre and post self-efficacy surveys.

| Variable | Description | Pre | Post |
| --- | --- | --- | --- |
|  | Total participants | 105 | 105 |
| Gender | Female | 91 (86.7%) | 91 (86.7%) |
|  | Male | 13 (12.4%) | 13 (12.4%) |
|  | Other/Prefer not to say | 1 (1.0%) | 1 (1.0%) |
| Age, years | Mean (SD) | 43.2 (14) | 43.8 (14) |
| Missing age | n (%) | 4 (3.8%) | 5 (4.8%) |
| Aged care experience, years | Mean (SD) | 9.4 (8.1) | 10.4 (8.6) |
| Missing aged care experience | n (%) | 3 (2.9%) | 5 (4.8%) |
| Staff role | Personal Care Worker | 75 (71.4%) | 75 (71.4%) |
|  | Enrolled Nurse | 6 (5.7%) | 5 (4.8%) |
|  | Registered Nurse | 21 (20.0%) | 22 (21.0%) |
|  | Other | 3 (2.9%) | 3 (2.9%) |
| Home | Home 1 | 8 (7.6%) | 8 (7.6%) |
|  | Home 2 | 4 (3.8%) | 4 (3.8%) |
|  | Home 3 | 13 (12.4%) | 13 (12.4%) |
|  | Home 4 | 9 (8.6%) | 9 (8.6%) |
|  | Home 5 | 9 (8.6%) | 9 (8.6%) |
|  | Home 6 | 7 (6.7%) | 7 (6.7%) |
|  | Home 7 | 14 (13.3%) | 14 (13.3%) |
|  | Home 8 | 10 (9.5%) | 10 (9.5%) |
|  | Home 9 | 14 (13.3%) | 14 (13.3%) |
|  | Home 10 | 6 (5.7%) | 6 (5.7%) |
|  | Home 11 | 11 (10.5%) | 11 (10.5%) |

**Table A4.** Matched analysis - Mixed model regression estimates for job related self-efficacy.

| Parameter | Estimate | Standard Error | 95% CI | p-value |
| --- | --- | --- | --- | --- |
| (Intercept) | 38.15 | 0.81 | 36.60 to 39.70 | <0.0001 |
| Post-intervention | 0.36 | 0.45 | -0.52 to 1.26 | 0.4319 |
| Age (+10 years) | 0.03 | 0.40 | -0.74 to 0.79 | 0.9498 |
| Gender: Male | 1.13 | 1.35 | -1.45 to 3.70 | 0.4060 |
| Gender: Other/Prefer not to say | 5.80 | 5.43 | -4.59 to 16.14 | 0.2875 |
| Aged care experience (+10 years) | 0.04 | 0.70 | -1.30 to 1.37 | 0.9593 |
| Staff role: Enrolled Nurse | -0.08 | 2.05 | -3.98 to 3.87 | 0.9706 |
| Staff role: Registered Nurse | 0.56 | 1.11 | -1.55 to 2.66 | 0.6156 |
| Staff role: Other | -2.89 | 2.66 | -7.96 to 2.17 | 0.2795 |

**Table A5.** Matched analysis - Mixed model regression estimates for team related self-efficacy.

| Parameter | Estimate | Standard Error | 95% CI | p-value |
| --- | --- | --- | --- | --- |
| (Intercept) | 25.85 | 1.36 | 23.26 to 28.44 | <0.0001 |
| Post-intervention | -0.57 | 0.59 | -1.74 to 0.58 | 0.3384 |
| Age | -0.03 | 0.03 | -0.10 to 0.03 | 0.3580 |
| Gender: Male | 0.71 | 1.09 | -1.38 to 2.80 | 0.5175 |
| Gender: Other/Prefer not to say | 4.76 | 4.94 | -4.73 to 14.20 | 0.3370 |
| Aged_care_time_years | 0.02 | 0.06 | -0.08 to 0.13 | 0.6660 |
| Staff role: Enrolled Nurse | 1.23 | 1.81 | -2.21 to 4.70 | 0.4981 |
| Staff role: Registered Nurse | 0.19 | 0.91 | -1.54 to 1.92 | 0.8329 |
| Staff role: Other | 0.77 | 2.12 | -3.26 to 4.80 | 0.7182 |

**Table A6.** Summary of semi-structured interview findings mapped to i-PARIHS.

| **Domain** | **Sub-Domain** | **Supporting Quotes** |
| --- | --- | --- |
| **Innovation** | **Relative advantage** | Yes so the bladder scanner we did use it .They (GPs) normally just want us to call QAS for them to do a mobile one [CM_2_Home_4] |
|  |  | They see the hospital as the better option, but in fact we are the better option at that end of life [RM_1_Home_1] |
|  |  | We don't really have doctor residing or like regular doctor visiting every day. So there's a little bit of difficulty contacting doctor and follow up on residents condition. We got the vital signs monitor and bladder scanner so it was more helpful to follow up on. We were using the equipment before we contact the doctor so we were like able to provide all the information in one go. [CF_4_Home_7]. |
|  |  | Yeah, they really like the vital signs monitor because we have a few residents who always like kind of have a fall then we were using the vital signs when they tell them to check their vital signs pretty regularly, which was really good and we have some residents with the catheter that are then they sometimes have problem with it, that it was really good to check if there's any built up in the bladder or something. So we didn't really have to send them to the hospital before we check them out. [CF_4_Home_7] |
|  |  | I think it's a good idea, especially like I think that age care industry, like not just our facility. Well, including our facility, but the whole industry. I know some people who work at different facilities and it seems like aged care are getting a lot of new grads and so I think like, something like EDDIE+ training would be good, it would have definitely helped like that with their confidence in like critical thinking. [CF_2_Home_10] |
|  | **Clarity** | everyone thought the concept was good and we were all really excited [CF_2_Home_10] |
|  | **Degree of fit** | Yes, that's what like I’m thinking you know it's changing all the time, policy changes and you know the staffing situation changes all the time. So anything can help the rest of the staff and to understanding you know deterioration or also provide quality of life. I think definitely personally from myself I think should be continued. [CM_3_Home_6] |
|  |  | It’s yeah, I think giving our nurses the tools to be able to assess and manage complex health within the home is a brilliant idea, we just don’t have the resources to get there. [RM_4_Home_11] |
|  |  | I think the theory behind it is great, and the education was great, the equipment fit in really well it was just that on the floor it’s not working at the moment. [RM_4_Home_11] |
|  |  | Good for new staff. Topics (scenarios and markers of deterioration) are things we see every day in aged care [CF_1_Home_1] |
| **Recipients** |  |  |
|  | **Values, beliefs, and motivation** | So, I think the actual program itself is brilliant, I think it was really great. [RM_1_Home_1] |
|  |  | I think as an ongoing thing, there’s always gotta be education, especially on those subjects [RM_1_Home_1] |
|  |  | we’re a lot better for having EDDIE+ then for not having it. That’s what I think. [RM_1_Home_1] |
|  |  | I've been working in aged care for so many years and sometimes some things change. But I know, but I don't recognize, that's a deterioration so it's sort of this program reminds me. [PCW_1_Home_6] |
|  |  | We had positive feedback and the carers (PCW), I'm not too sure how they got the program and how they, you know, welcomed the program. But of course, our registered staff, they were actually really happy about learning new things and getting to know to use the bladder scanners and like the ECG's and things like that. [RM_5_Home_6] |
|  |  | Empowered the registered nurses more, because being in aged care they get a bad rep (reputation) cause you know, just the stigma I guess from aged care nursing compared to hospital nursing so I think it gave them more confidence. [RM_2_Home_7] |
|  | **Self-efficacy, skills, and knowledge** | The high RN turnover, it's probably very vital to keep them in the loop and upskill them. [RM_5_Home_6] |
|  |  | You can have that you can have a better system in place but if they’re not implemented in a knowledgeable way then it kind of can be all for nothing. [Other_3] |
|  |  | I think everyone here and the practice manager we have, we’re sort of all for that sort of intervention to increase people's capability. And it goes beyond that, it's an important point to recognize that it goes beyond just improving the care actually improves peoples job satisfaction. [Other_3] |
|  |  | We had quite a few times the registered staff used the scanner even like that, we could avoid hospitalizations. [RM_5_Home_6] |
|  |  | We didn't really have to send them to the hospital before we check them out. [CF_4_Home_7] |
|  |  | Used bladder scanner on resident who had history of urinary retention and found it really helpful. [CF_1_Home_1] |
|  |  | External Facilitator came back, maybe, I don't know, six weeks or something after all the initial training and he noticed that no one had actually used the obs machine. [CF_2_Home_10] |
|  |  | We had a fellow only not that long ago that had some retention, so we were able to do a scan and the guys were able to pop an IDC in (catheter) and that actually did stop him from being transferred to hospital. [RM_2_Home_7] |
|  |  | This is my frustration when I was working in clinical education is that we can go and talk to them and they’re all really enthusiastic but when they hit the floor, they cant picture how to actually utilise the information in practice and I think that’s a flaw with any kind of education going into our facilities – the inability of staff to actually take their learning and apply it in practice. [Other_1] |
|  |  | The culture in aged care that they’ve all been I guess mentored into where you’re task focused and you’ve got a lot of work to do in a single shift and you put your head down and do it. So, there’s not a lot of reflection of practice and there’s not a lot of thought about the global picture which is what you need when you’re looking at deterioration. So it’s a lot around culture but it’s a lot around lack of skill and experience and inability to take this information and reflect on it and add it to your practice. [Other_1] |
|  |  | Their clinical leadership, their clinical manager is not a very experienced RN themselves, so there’s no leadership from the top. [Other_1] |
|  |  | Learning new skills and ways to approach problems. [RN1335_5] |
|  |  | The best part of the EDDIE+ program is gaining knowledge about deterioration of the resident which will help to save them from several things such as fall, UTI and so on. [PCW1222_12] |
|  |  | It gave more knowledge about how to take care of elderly and also how to understand their behaviour and situation [PCW1367_11] |
|  | **Collaboration and teamwork** | A home is not a hospital and it doesn't have medical care available. To me it's super important because either way, if you can't, if you can get on to a GP, then the premise behind it is the nurse has got accurate information to give the GP to determine an outcome. And that to me is gold, right. If you can't even articulate what's wrong, that's not helpful. And also, if the if the GP is not available to take call once again when the ambulance arrives, when you're calling the ambulance, you've got real information. Those things are extremely valuable. [Other_2] |
|  |  | By having clear information and that's what proper equipment will do, it will give you an accuracy to go this is what I need to pass on. [Other_2] |
|  |  | After the education we can see and observe better rather than, you know, just passing by to other people, we can sort of identify, yeah, and talk to the nurse. Yeah, I think that was quite good because I often we see, but we don't really pay attention [PCW_1_Home_6] |
|  |  | My observation skills improved, you know, I go always go tell the nurses. Sometimes they don't like to hear it anymore, more job for them. But yeah, I think it's overall it's gonna improve because people can recognize more and if they know more, then they can sort of identify rather than, oh, I don't know. [PCW_1_Home_6] |
|  |  | After I introduced the all the communication tools from EDDIE+, care staff were more motivated and like encouraged to report it because they're happy to see what change is made and what like they noted and made the difference in residents condition. So they’re really on it. [CF_4_Home_7] |
|  |  | They're like better at reporting things now and they're a bit more conscious of, like picking up on changes and stuff. [CF_2_Home_10] |
| **Local Context** | **Mechanism for embedding change** | Brilliant idea, we just don’t have the resources to get there. [RM_4_Home_11] |
|  |  | It’s a great program, great in theory, training was great, equipment’s great, it’s just unfortunate that we can’t push it through more. [RM_4_Home_11] |
|  |  | I think the idea of EDDIE+ is fantastic and I really really want it to be successful but I think in terms of getting it to cement itself in our facilities, we need more than just the education, we need the mentoring and coaching. [Other_1] |
|  |  | It will be really beneficial and for us as well, we got the equipment so well we’ll be continue on those. But it was really helpful. [CF_4_Home_7] |
|  |  | From a quality and safety perspective, levels of workforce are a barrier, but then we have to have capability and knowledge and experience but that’s linked to the EDDIE+ project because you know, that's something that we don’t normally have the luxury of in residential aged care, compared to other parts of the healthcare sector. So anything that can help develop capabilities is important. Moving on from there then I guess we have support to identify deterioration and then also, policies, procedures and guidelines that. [Other_3] |
|  |  | It’s staff availability, it doesn’t matter how you, how you dress it up, if the staff are unable to attend the session or we have the turnover and lack of retention of staff it’s a problem. In some cases, we have 30% turnover of staff only from a clinical team between each quarter. So you may educate people on these particular equipment and identifying deterioration but then they leave and you get a new person in so yeah it’s maintaining the consistency. [Other_3] |
|  |  | I’m worried about I mean we’ve seen it over and over again with all sorts of things you know you get a home upskilled, it’s going great, then there’s a turnover of staff and its forgotten so I am meeting with our senior clinical nurse advisory group to make changes to our clinical deterioration guidelines, I’m working with our onboarding team to develop onboarding around clinical deterioration and the recommendations, so I’m hoping that those sort of strategies that we’re putting in place will actually support the continuation. [Other_1] |
| **Organisational** | **Staffing** | I do really like the program. I'm just disappointed that like it wasn't probably like from me like it wasn't done probably like well as I could have done it just with all the different roadblocks. [CF_2_Home_10] |
|  |  | The impact of staffing over the past two years, you know with the roster here, just for the PCW there’s over 135 vacant shifts in a two-week roster. [RM_3_Home_4] |
|  |  | I think the main issue would just be yeah the staffing. But with the EDDIE+ itself, I think it's pretty amazing. [CF_3_Home_3] |
|  |  | Even before COVID, the fact that the grant actually allowed back filling because part of the issue is not about the cost of education, it's about getting people off the floor to attend education. [Other_2] |
|  |  | Overall, even like as a PCW, we see the problem and we informed them this, and, you know, this is what's happening but sometimes because of nurses workload, they can’t always follow up. I think that was the problem, it's not because of the EDDIE+ program, it's just in general the work load. [PCW_1_Home_6] |
|  |  | I've been here for quite some time as some of the other girls, but we're getting less and less because they're leaving either through age or they've had enough they're just sick and tired of working hard and long and the body is given out. [PCW_2_Home_4] |
|  |  | We’ve had a staffing crisis but having an RN in that role you know as much as you backfill a role, the demand was just too great that they sometimes got pulled into having to not deliver the service, so they are factors. I’ve been in aged care a long time and I’ve never known a staffing crisis like the one we’ve been in you know. [RM_1_Home_1] |
|  |  | We discovered everything is just getting so tight so it's like even the nurses are overworked and then you know even the carers sometimes when we are short staffed. [PCW_1_Home_6] |
|  |  | I think industry wide we don’t have staff and yeah I think it started with covid and um I think we thought we’d get back to normal by now and it just hasn’t so I’m not actually sure if its covid related. [RM_4_Home_11] |
|  |  | They’re always on double shifts and working PCW shifts most the time as well so, the basic cares are getting done and that’s kind of where its finishing. [RM_4_Home_11] |
|  |  | I actually had one of my nurses rang me late last night they wanted to send someone to hospital and I’m trying to work out why and if there’s anything we can do and you can just hear the phone in the background going mad, we’re short staffed and there’s people yelling and stuff and it’s just like nah call QAS (Queensland Ambulance) and get them out because you don’t have the resources right now to do that. [RM_4_Home_11] |
|  |  | When you go in lock down sometimes then you prioritize, the EDDIE does not come as a priority because your priority comes as keeping the staff, then managing the outbreak. [RM_5_Home_6] |
|  |  | We don't have management long enough to do anything. Yeah, I mean, because they're sitting on that seat and then they're gone and change then change, so every time somebody either starts making inroads for whatever reason, they're gone so then it's back to the start. [PCW_1_Home_6] |
|  |  | I've seen staff that have left this company that ended up there were the bosses, they were lifetime managers, and they moved on to other areas and then they went up the promotional stair. [PCW_1_Home_6] |
|  |  | There was some training stuff organized, but people just weren't starting to come to it, trying to drag people off the floor became an issue. [QUT_project_team _1] |
|  |  | They just pulled them off the floor [for training] or said, are you coming on your day off or they tacked on at the end of this shift [QUT_project_team _1] |
|  | **Culture** | I think they just get into a pattern of if they do identify anything the resident gets sent to hospital [RM_3_Home_4] |
|  |  | People just didn't rock up to try and I was sitting there twiddling my thumbs I think for one day and I didn't have one person and I had four or five training sessions booked. [QUT_project_team _1] |
|  |  | Their attitude was like oh we've got to go to training? You know I’ve got enough to worry about. [QUT_project_team _1] |
|  |  | There is no reward there's no incentive, the morale, it's disgraceful. When people plain repeat, it's like this everywhere, I don't work everywhere, I only work here, this is my workplace, this is where I am working short staffed, the frustration, the lack of support, the lack of morale building, the lack the lack. There is no for the people that go above and beyond. Nothing. [PCW_2_Home_4] |
|  |  | I feel sorry for some of the women because there are genuine people that love their job going above and beyond to make the residents life and their work place a better place to walk in and for them to attend. Yeah, but when you get no support, like seriously?. [PCW_2_Home_4] |
|  |  | There’s quite a degree of anxiety among the staff and you know we don’t want to be looking after this here, it’s scary, let’s get them off to hospital. [Other_1] |
|  |  | The managers there the clinical manager and RN were excellent and really supportive [QUT_project_team _1] |
|  | **Learning Networks** | I’d really like to get hold of all the education modules and put them up on our learning management system so that we’ve got, we can say to staff hey go and have a look at this or we can actually run education sessions where we’ve got the staff in the room then we can talk it through and say what are you gonna do in practice for next time you encounter this. [Other_1] |
| **Health System/Wider Level** | **Royal Commission** | The royal commission and all these other things that have been out there haven’t helped. [RM_1_Home_1] |
|  |  | I think the families don’t always know that we’ve got such fantastic equipment, that we’ve got trained staff you know I wish that they would believe us, like you know what they say on TV, they don’t know this, they’re not trained. [RM_1_Home_1] |
|  |  | So that was already coming out of the royal commission. They had recognized that you needed higher skilled workforce to get better resident outcomes. [Other_2] |
|  |  | One of the challenges that I see, you know, I've recruited graduate nurses for decades and a graduate goes into a clinical environment with a novice skill set. And through a graduate program. But you know where most of the learning occurs, it's actually from the nurses around you that are experienced, right? You learn something, they show you something. It's that mentorship and job experience. That's what's missing in aged care because a graduate in a hospital has got a buzzer on the wall that they can walk one meter. If they're savvy enough to pick up some sort of clinical deterioration they go and get a senior nurse and if it's a true emergency, help is there and then they learn from that. The problem in aged care is these people are graduates and they might be the only RN. Yeah, there is no mentorship. And if you look at Patricia Bena's theory of novice to expert, it's educational learning plus job training and mentorship and that's the piece that is missing. [Other_2] |
|  |  | Not just in (Home name), in aged care generally we have, in terms of our registered nurse cohort, we have very few experienced, skilled, senior nurses and we have a lot of less experienced, less skilled, less, I don’t know how to say it politely, we do get the new grads who don’t get on to grad programs so we’re starting from a lower base point. [Other_1] |
|  |  | We do seem to be getting a lot of novice practitioners coming into the aged care sector, certainly into (Home name) and that's a major challenge. Ideally we’d have a good clinical nurse here for 10+ years and they’re very experienced. [Other_3] |
|  | **Government Funding** | The federal government gave residential care grants to get an infection prevention champion lead, and the solution was but here’s money, here's money for them to go and do the course and to backfill them. The issue we had was you could not get even like in theory, and that was the same with EDDIE+ let's ring up an agency, get an agency in to get on the floor while we backfill like we educate our staff. It was the same thing with the infection control person as well. The education was brilliant. The government had given us money. There was no workforce to backfill. So that's been a particular challenge, that, in theory, is if you can get people in to replace, but there wasn't even that. [Other_2] |
| **Facilitation** | **Internal** | Maybe more hands on like more as in more I know that's kind of what the facilitators were, but maybe that's an idea. Like instead of having home facilitators that are getting always pulled away and you know busy with other things like maybe an idea would be to have an external person to come in and run through scenarios and stuff like. That way you know, I was getting, work this shift instead or do this or, you know. It could be hard to juggle everything. So yeah, if it was like an external person.[CF_2_Home_10] |
|  |  | To have an external person maybe could come in two days a week for six weeks or you know, something like that, as an example. That way and that way like it's a constant reminder of like ohh that's right, we've got that equipment or you know it rather than just having me or someone who's here all the time, right? Instead of sort of, as I said, pulling people off the floor to do this. If there was someone that could sort of facilitate it externally, that would be helpful. Yeah, I think, yeah. I think that's probably the biggest right point that I like wanted to make was. Yeah, I think that would be more helpful. [CF_2_Home_10] |
|  |  | Not only are they working here, they have another facility to go to. So they're up, you know, late, so they're missing their handover and then they don't know where the staff that has arrived on time, where they are on the floor, so they're walking around waste and more time to see who they have to do and where they have to go because they don't know how to the residents cares and needs have changed. So then again, you have another break down there. [PCW_2_Home_4] |
|  |  | It’s dependent on the champion but, you know Name and who did the delivery of the education was brilliant. [RM_1_Home_1] |
|  |  | The first person didn’t vary her days, so she was stuck on the same days which meant she kept getting the same people. [RM_1_Home_1] |
|  |  | When you’re an educator you still have to deliver it in a very energetic and interesting way and she didn’t she would, she had a very sedate sort of personality, so there wasn’t much engagement from the others (staff). [RM_1_Home_1] |
|  |  | If we can link with real resident real scenario cases, I think we can help the staff understand better. [CM_3_Home_6] |
|  |  | just go through you know, there's the resident of the deteriorating. Now what? What have you seen? You know what? What, what does that tell you?. [CM_3_Home_6] |
|  |  | I couldn't do a lot of extensive training due to COVID lockdown because we were under the lockdown from January to April, so it was pretty hard, but still we were using the machine really effectively and the staff was happy with the communication tools. [CF_4_Home_7] |
|  |  | I didn't find it manageable, but it's not because of what was expected of me it's because like I was constantly getting pulled away from doing it. It's like more our issue than it is yours because I do think that if I was given like heaps of adequate time, it would have been fine. [CF_2_Home_10] |
|  |  | We actually had to pick a champion to start off with and then we picked one of our new RNs Name, and she really excelled it in, she actually went really really good and it gave her more confidence actually, she’s a bit on the quiet side but I knew the level of potential there. [RM_2_Home_7] |
|  |  | So I was one of the facilitators, ohh and also I unfortunately was on leave for a large chunk of the rollout as well, it just like it wasn't planned very well and there was no one else to be a facilitator. We also we did have another nurse called Ella who was going to be a facilitator, but she actually ended up like resigning. OK, so it all just kind of you know, it fell down. [CF_2_Home_10] |
|  |  | As soon as we said that she was going to be the facilitator everyone's eyes lit up. [QUT_project_team_1] |
|  |  | I think all of the ones that also had a good sort of personality traits also had seem to have respect in the clinical sense. [QUT_ project_team _1] |
|  | **External** | I think the guys who did the education were very approachable and that made it easier just with their approach and everything made it easier on the staff and that worked really well. [RM_2_Home_7] |
|  |  | I think everyone is happy with the education that we received. [CM_2_Home_4] |
|  |  | I did enjoy the interaction and I did feel, what’s that chaps name? Name, I felt he listened, and he knew what we were talking about. [PCW_2_Home_4] |
|  |  | Appreciated that the training they delivered was interactive and not just talking to a group of people for an hour. [CF_1_Home_1] |
|  |  | They were pretty supportive they were doing the, like, follow up phone calls every week when I did it, check in forms and there were like, quick, like providing the information if I need it via the email and online and even post. So I was pretty happy with it. [CF_4_Home_7] |
